# ERANET JTC 2011: Submission and Activation of an International Academic Translational Project in Advanced Breast Cancer. Experience From the ET-FES Study

**DOI:** 10.3389/fmed.2021.817678

**Published:** 2022-01-13

**Authors:** Manuela Monti, Tom Degenhardt, Etienne Brain, Rachel Wuerstlein, Alessandra Argusti, Matteo Puntoni, Gian Andrea Rollandi, Davide Corradengo, Luca Boni, Harun Ilhan, Oriana Nanni, Javier Cortes, Alejandro Piris-Gimenez, Arnoldo Piccardo, Massimiliano Iacozzi, Federica Matteucci, Valentina Di Iorio, Jean Louis Alberini, Carolien Schröder, Nadia Harbeck, Alessandra Gennari

**Affiliations:** ^1^Unit of Biostatistics and Clinical Trials, Istituto di Ricovero e Cura a Carattere Scienteifico Istituto Romagnolo per lo Studio dei Tumori “DinoAmadori”, Meldola, Italy; ^2^Breast Center, Department of Obstetrics and Gynaecology, Ludwig Maximilians University Hospital of Munich, Munich, Germany; ^3^Department of Clinical Research and Medical Oncology, Institut Curie-Hopital “René Huguenin”, Saint-Cloud, France; ^4^Clinical Trial Research Unit, Ente Ospedaliero Galliera Hospital, Genoa, Italy; ^5^Clinical Trials Unit, Istituto Nazionale per la Ricerca sul Cancro Istituto Scientifico Tumori, Genoa, Italy; ^6^Die Radiologie (Centre for Radiology, Nuclear Medicine and Radiotherapy), Munich, Germany; ^7^Vall d'Hebron Institute of Oncology (VHIO), Vall d'Hebron Barcelona Hospital Campus, Barcelona, Spain; ^8^Department of Nuclear Medicine, Ente Ospedaliero Galliera Hospitals, Genoa, Italy; ^9^Nuclear Medicine Department, Centre “Georges-François Leclerc”, Dijon, France; ^10^Department of Medical Oncology, Dutch Cancer Institute Nederlands Kanker Instituut - Antoni Van Leeuwenhoek, Amsterdam, Netherlands; ^11^Department of Translational Oncology, University of Eastern Piedmont, Novara, Italy

**Keywords:** ERANET, academic, regulatory in Europe, radiopharmaceuticals, PET

## Abstract

**Background:** Academic research is important to face unmet medical needs. The Oncological community encounters many hurdles in setting up multicenter investigator-driven trials mainly due to administrative complexity. The purpose of a network organization at a multinational level is to facilitate clinical trials through standardization, coordination, and education for drug development and regulatory approval.

**Methods:** The application of an European grant foresees the creation of a consortium which aims at facilitating multi-center academic clinical trials.

**Results:** The ERA-NET TRANSCAN Call 2011 on “Validation of biomarkers for personalized cancer medicine” was released on December 2011. This project included Italian, Spanish, French and German centers. The approval process included Consortium constitution, project submission, Clinical Trial Submission, and activation on a national level. The different timescales for submitting study documents in each Country and the misalignment of objections by each Competent Authority CA, generated several requests for changes to the study documents which meant amendments had to be made; as requested by the 2001/20/EC Directive, the alignment of core documents is mandatory. This procedure impacted significantly on study activation timelines. Time to first patient in was 14, 10, 28, and 31 months from the date of submission in Italy, France, Spain, and Germany, respectively. Accrual was stopped on 22nd January 2021 due to an 18F FES shortage as the primary reason but also for having exceeded the project deadlines with consequent exhaustion of the funds allocated for the project.

**Conclusions:** Pharmaceutical companies might be reluctant to fund research projects aimed at treatment individualization if the approval for a wider indication has already been achieved. Academic trials therefore become fundamental for promoting trials which are not attractive to big pharma. It was very difficult and time consuming to activate an academic clinical trial, for this reason, a study may become “old” as new drugs entered into the market. National institutions should promote the development of clinical research infrastructures and network with competence in regulatory, ethical, and legal skills to speed up academic research.

## Introduction

Trials to improve the knowledge on personalized therapy strategies are usually developed on large-scale populations. Therefore, funding is a difficult issue, as the unmet medical needs to better understand who is really benefiting from a drug with a broad indication, does not necessarily arouse the interest of the industry (1). As a consequence, there is an unmet medical need that could be addressed by independent academic research in particular multi-institutional, international translational research. It is of great interest to strengthen translational cancer research with the integration of basic, epidemiological, preclinical, and clinical research with the implementation and evaluation of interventions in prevention, diagnosis, prognosis, treatment, and care. Oncological clinical research community encounters many hurdles in setting up multicentre trials, particularly for Investigator-driven academic trial. The main issues concern the administrative complexity and heterogeneous clinical staff training and infrastructure support that often limit the opportunity to participate into international clinical trials. Efficient planning and performance of clinical research rely on the interplay among teams of different clinicians and other components such as ethical committees, national and local authorities, promoter and drug manufactories, patient association, as well as hospital administration. Joining forces within multinational project applications and more interdisciplinary projects will be necessary to realize the full potential of the increasing number of developments for theragnostic applications. The scope of a network organization at a national level is to facilitate the effective use of molecular imaging in clinical trials through standardization, coordination, and education for drug development and regulatory approval. The Italian network model could be transferred to an European level to facilitate the participation of all network centers into Investigator-driven non-academic International multicentre clinical trials. Molecular imaging with PET is a rapidly emerging technique. In breast cancer patients, more than 45 different PET tracers have been or are presently being tested. But regretfully so far, only [^18^F]-FDG PET has been incorporated into breast cancer guidelines. PET tracers will likely allow better breast cancer patient selection for the right treatment. However, for proof of the clinical relevance of the tracers, especially for analysis in a multicenter setting, standardization of the technology and access to the novel PET tracer are required. Funding for such an approach has largely been lacking. The ERA-NET TRANSCAN call aims at combining translational cancer research funding programs in 19 Member States and Associated Countries. TRANSCAN will concentrate translational research resources and will provide relevant financial support to address large scale problems that will be relevant for the improvement of translational cancer research in every Member State and possibly overall in Europe. TRANSCAN will identify opportunities for coordinated translational research, and will thus contribute to the development of a coordinated funding research policy shared by European countries. The activation of an international, non-profit clinical trial supported by the ERA-NET (Aligning national/regional translational cancer research programmes and activities) and funded by the European Commission requires specific timelines according to the EU rules. This paper describes the complexity of activating an international study within the ET-FES TRANSCAN project in 4 EU countries (France, Germany, Italy, and Spain).

## Materials and Methods

### Project Selection for Funding According to the Type of Call

The call ERA-NET on Translational Cancer Research (TRANSCAN) First Joint Transnational Call for Proposals (JTC 2011) on: “Validation of biomarkers for personalized cancer medicine” was available for a proposal submission on 10th of January 2012. The Chief Investigator (Italian PI) decided to submit a proposal on an interventional clinical trial for breast cancer patients: Early prediction of efficacy of endocrine therapy in breast cancer: pilot study and validation with [^18^F]-fluoroestradiol (FES) PET/CT - ET FES study. The availability of this non-invasive functional test to assess the endocrine responsiveness in the individual patient with multiple breast cancer metastasic sites represents an interesting option. The availability of new techniques such as molecular imaging with [^18^F]-FES CT/PET offers the opportunity to improve the ability to predict the probability of response to endocrine therapy. To be compliant with the call, a consortium was created with the purpose of implementing a network of clinical centers, each including Medical Oncology Unit and Nuclear Medicine Unit in order to optimize the multidisciplinary approach needed to perform this clinical trial for what concerned the clinical aspects, regulatory framework, logistic and technical aspects. The project coordinator implemented standard operating procedures (SOPs) and transferred them to the other participant partners, to set up an international EU network for this translational imaging project. While applications were submitted jointly by the coordinator of this group at an EU level, each Country was funded by the responsible national funding organization; funding was available by each national/regional funding organization according to their specific regulations. The funding rate within the call ranged up to a maximum of 100% of the funds requested, according to national/regional rules. Funding was granted for a maximum of 3 years according to national regulations. Applicants contacted their national/regional funding organizations prior to submitting a proposal to verify their eligibility, the eligible costs, and the potential budget available. Depending on the time needed for the administration of granting funds to the respective national/regional research groups, individual projects of a research consortium were expected to start between March and April 2013. Only if selected for funding, the project coordinator/promoter of the study, could have started the submission process of the clinical study in all Countries involved in the consortium taking into account that the clinical trial authorization process is on a national basis.

### Ethics Approval Statement

Ethical approval was obtained from IRST and Romagna Ethics Committee (CEIIAV) on 22nd January 2014 (Prot. 426/I.5/242). It was conducted in accordance with the 1964 Helsinki Declaration and its later amendments and with Good Clinical Practice (GCP) guidelines. Written informed consent was obtained from all individual participants included in the study.

## Results

### Approval Process for the European Project (Consortium Constitution and Project Submission for EU Approval)

The ERA-NET on Translational Cancer Research (TRANSCAN) Joint Transnational Call 2011 (JTC 2011) for European Research Projects on “Validation of biomarkers for personalized cancer medicine” was released in December 2011. In order to meet the call requirements, this study had to be international, so it was decided to include Spain, France and Germany. The Project Coordinator (PI) contacted the reference Nuclear Medicine Departments of each state and presented the project to them. The PI also looked for a Company authorized to produce the experimental radiopharmaceutical [^18^F]-FES. These relations made it possible to set up the project and prepare the documentation for submission to the European call.

### Consortium Constitution

The Consortium provided high competence and expertise related to the project's scope for what concerned scientific, technological and regulatory areas. In particular, it consisted of five partners from four European countries and represented in all key actors in a balanced way, in a trustworthy domain and addressed the project's key topics. A collaborative network was newly established among the Nuclear Medicine physicians, sharing all aspects related to the [^18^F]-FES with particular emphasis on logistical and technical aspects. The Project Coordinator and Sponsor was E.O. Ospedali Galliera, Genoa, Italy; the other partners were Istituto Scientifico Romagnolo per lo Studio e la Cura dei Tumori, IRST IRCCS (Italy), Vall d'Hebron Insititute of Oncology (VHIO, Spain), Breast Center, Dept. OB&GYN, LMU University Hospital, Munich (Germany) and Institut Curie—Hôpital René Huguenin, Department of Medical Oncology (France). All these partners are medical Institutions of excellence, particularly dedicated to biomedical and health system research with its focus on cancer. Advanced Accelerator Applications (AAA), a French radiopharmaceutical company operating in the diagnostics and therapy field of Nuclear Medicine, located in the Technology Park (Ain, Saint GenisPouilly) was also part of the consortium and developed [^18^F]-FES for the ET-FES TRANSCAN project.

### Project Submission

After the letter of intent had been approved, the final ET-FES application was uploaded on 2nd July 2012. The proposal was approved for funding by email on 11th October 2012 by the ERANET Committee and TRANSCAN Secretariat. No additional information on the Scientific Evaluation Committee's judgments or extent of funding overall and in the different countries was provided. The Secretariat suggested that all principal investigators contacted their respective national funding organization in order to start the (national) negotiation process. The official starting date for the project was the 30th of June 2013, 8 months after the expected date. This was due to the extensive and time consuming negotiations between E.O Ospedali Galliera (Project Coordinator, PC), the Italian Ministry of Health and Liguria region as legal regional representative. In October 2013 the PC/Sponsor of the ET-FES study received the final approval from Liguria Region allowing the start of the approval procedures, which was 4 months after the official start of the project. The first scientific report was due in December 2013 and required a summary of the activities performed on the project on the 1st year, with economic justification. The Slovak Academy of Sciences (SAS) as the TRANSCAN partner responsible for the monitoring of JTC-1 received the report in time.

### Clinical Trial Submission and Activation

The study was configured as an academic, interventional clinical trial; the radiopharmaceutical ([^18^F]- FES) used for PET/CT must comply with the legislation on drugs; current legislation on Clinical Trials must be observed (European directive 2001/20/EC declined in the various Member States, Decree n° 211 for Italy). [^18^F]-FES is a radiopharmaceutical that is not easily produced and it has no Marketing Authorization yet. On 29th September 2013, clinical ET-FES study protocol was finalized and approved by all the involved partners.

None of the countries could begin the study until approval by the reference Ethics Committee (EC)/Institutional Review Board (IRB) had been obtained and until the local regulatory requirements complied with the national competent authorities. The Sponsor provided each Country with the core documents (final protocol, Investigator's brochure, Investigational Medicinal product dossier, subject information sheets, consent forms) (2) and all other relevant study documentation for local required submissions. Patient Informed Consent was completed and translated into the different languages. A trial insurance policy was stipulated. The principal investigators and the Sponsor ensured that the study was conducted in full conformance with the 1964 Declaration of Helsinki principles and with the laws and regulations of the country in which the research was being conducted, whichever afforded the greater protection to the individual. The study had to fully adhere to the principles outlined in “Guideline for Good Clinical Practice” ICH-E6 Tripartite Guideline (January 1997) and with national laws. For the Study conducted in the EU/EEA countries, the Principal investigator would ensure compliance with the EU Clinical Trial Directive (2001/20/EC), ICH GCP and EU Data Protection Directive (95/46/EC).

In parallel, the development process of [^18^F]- FES, according to Good Manufacturing Practice (GMP), started in January 2013 and it was completed in November 2013. All the logistics for tracer shipping and delivery had been set up. A Financial contract was put in place between AAA and the project coordinator in order to define the budget and timing for study drug supply. Study drug would be provided to all the Sites (3) from French laboratories. [^18^F]-FES was defined as Investigational Medicinal Product (IMP). The current “Clinical Trials Directive” defined the requirements for authorization of manufacturing an IMP, which includes applying Good Manufacturing Practices (GMP). Within this regulatory framework, also Good Clinical Practices (GCP) for conducting clinical trials were mandatory, stating responsibilities, requirements, and structure of clinical trials (ICH GCP). The documentation package for a clinical trial application included both information on the IMP as well as on the conduct of the clinical trial itself. All the information concerning the radiopharmaceutical to be used in the trial was included in the Investigational Medicinal Product Dossier (IMPD) (4, 5). The information in the IMPD has to be given in a standardized way, which is based on the so called Common Technical Dossier (CTD) format, which is also used in applications for marketing authorization. The IMPD addressed chemical and pharmaceutical properties covering the quality of a new release criteria, analytical procedures and their validation; in the second part of the IMPD, information on the safety and efficacy of the IMP should be provided (3). Regarding the quality part of IMPD, the Company sent the quality dossier directly to Competent Authorities (CAs) in order to maintain the confidentiality of [^18^F]- FES production data; therefore the objections from CA would be communicated to the Company only. This process certainly makes the authorization process more complicated as the Sponsor (different from the Company) is not directly involved in the quality IMPD submission and must wait for approval of this part which is not under his direct control and responsibility. The Investigational Medicinal Product Dossier (IMPD) and the Investigator's Brochure (IB) have to be finalized and provided for submission to the CAs by the Clinical Trial Sponsor. Under the Directive, Clinical trial application has to be approved on a national level both by EC/IRB and national CA within defined timelines according to Directive. National radioprotection competent authorities were also involved and there was a very time- consuming procedure related to the high heterogeneity between Countries.

Each National Principal Investigator submitted the study to its own EC/IRB and CA. The ET-FES study was submitted in Italy to the Coordinating EC and CA in December 2013 and it was approved by Coordinating EC in January 2014 while final AIFA approval came on 13th August 2014 and Ministry of Health (radioprotection Office) approval in October 2014. In February 2015 the submission package was sent to France EC/IRB and CA and the final approval came on 01st Jul 2015. On 24th November 2014 the submission package was sent to Spanish EC/IRB and CA and the final approval was released on 08 August 2015 (conditioned approval) by AEMPS. On January 8th 2015 submission package was sent to Germany EC/IRB and CA but the final approval came only in March 2017 (re-submission was required to avoid a refusal due to quality IMPD concerns).

**Figure 1:**
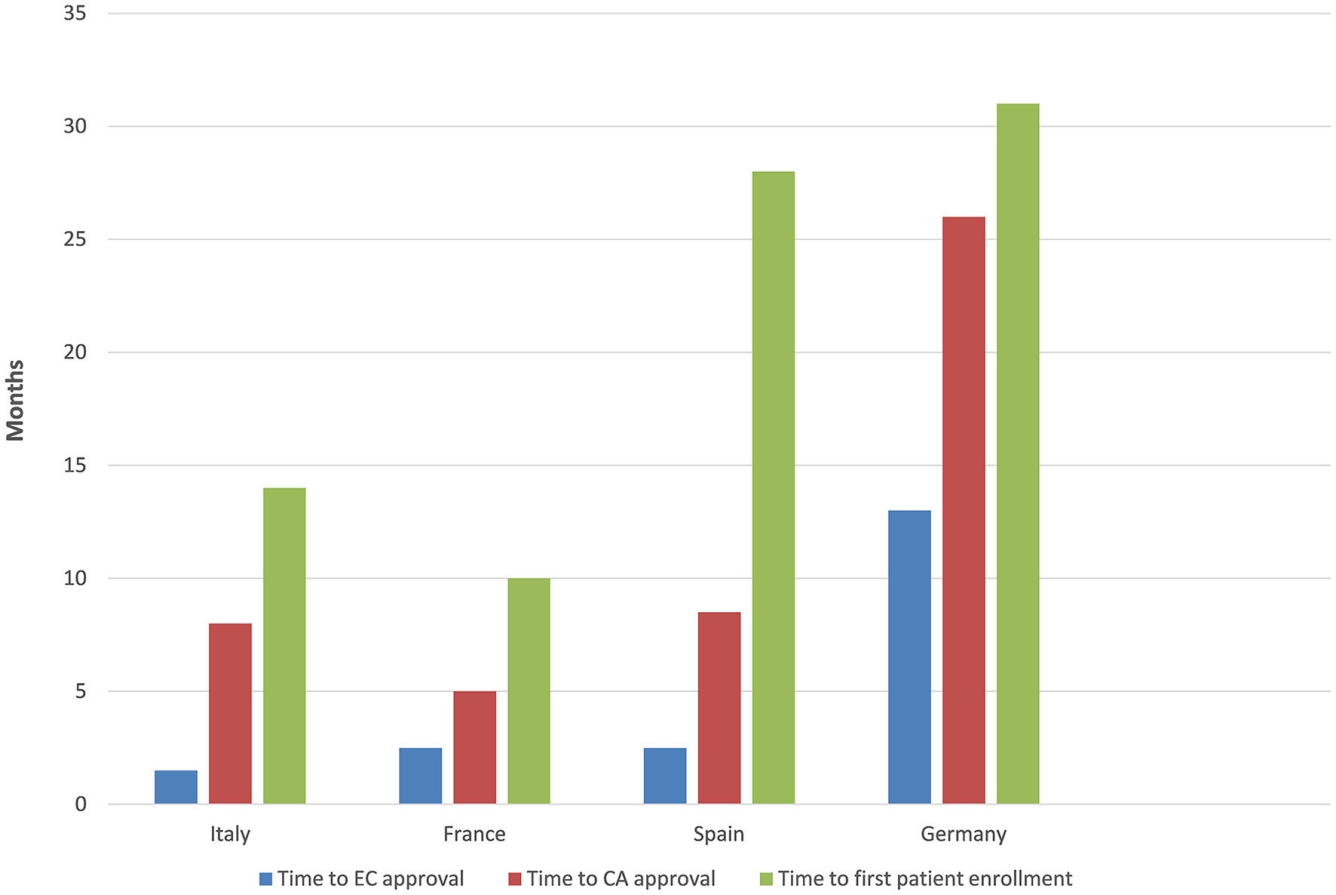
Timelines.

**Figure 2:**
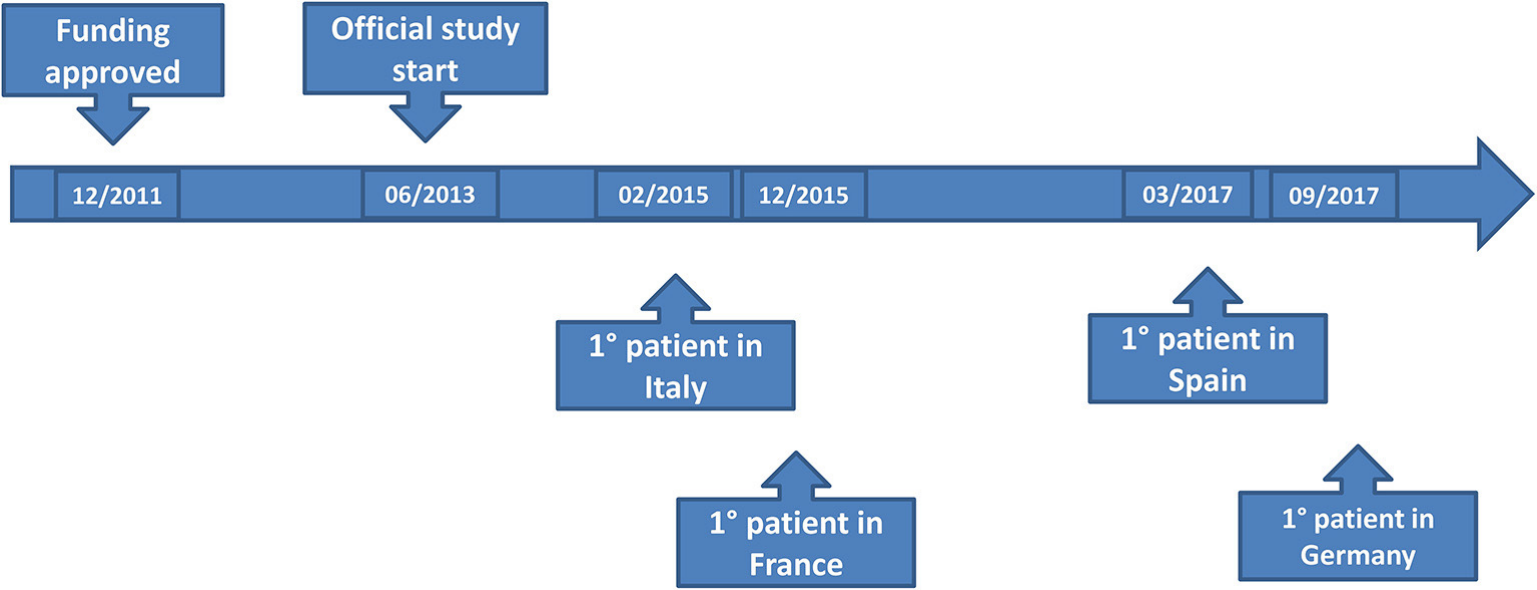
Sites activation.

The time to EC approval was 1.5 months for Italy, 2.5 months for France, 2.5 months for Spain, and 13 months for Germany (due to amendment submission). The time to CA approval was: Italy 8 months, Spain 8.5 months, France 5 months, and in Germany 26 months (due to re-submission) (see Table 1 and Figure 1). Overall, no ethical objection was raised by any of the ECs; some minor clinical and methodological issues were raised from the EC/IRB in Germany and Spain. Issues from the CAs were raised in all countries, except France (12 queries in Italy, 21 in Spain, and 34 in Germany), mainly regarding quality aspects of [^18^F]-FES IMPD (see Table 2). At Sponsor level, the time to the final agreement signature with the [^18^F]-FES manufacturing company required 13 months. After finalization of all contracts and approval by EC and AC, the first patient was enrolled on 6th February 2015 in Italy: this was 14 months after EC submission and 20 months after the official start of the ET-FES project, as set up by the Italian Ministry of Health and communicated to the Joint Call Secretariat (JCS). The time to first patient in was 10, 28 and 31 months from the date of submission in France, Spain, and Germany, respectively (see Figure 2).

**Table 1:** Timelines.

**Country**	**Time to** **EC approval** **(months)**	**Time to** **CA approval** **(months)**	**Time to** **signed contract** **(months)**	**Time to 1st** **patient in** **(months)**	**Time from funding** **to 1st patient** **(months)**
Italy	1.5	8.0	13.0	14.0	20.0
France	2.5	5.0	9.5	10.0	30.0
Spain	2.5	8.5	18.0	28.0	59.0
Germany	13.0	26.0	27.0	31.0	60.0

**Table 2:** Objections by EC and CA.

**Country**	**Objections**	
	**Ethics committee**	**Competent authority**
Italy	-	12 (AIFA)
France	-	0 (ANSM)
Spain	Minor	21 (AEMPS)
Germany	Minor	34 (BFARM)

In particular, in Germany, the main reason for the delay was a difficult and time-consuming approach to get approval by CA for the study (6), which was already enrolling patients in Italy and France; Germany's CA raised several questions concerning the quality aspects (quality IMPD) of the tracer which had been approved to be used in the study in Italy, France and Spain. German CA concerns mainly addressed cold chemical precursor: according to CA request, it should be described and characterized to an extent which was usual for active substances in clinical trials. These changes had to be submitted as Substantial Amendment to the current IMPD and they took time to align the Country specific documentation also for Italy, France, and Spain.

The different timescales for submitting study documents in each Country and the misalignment of objections by each CA, have generated several requests for changes to the study documents with the consequent need to make Amendments; as requested by the 2001/20/EC Directive, the alignment of Core documents is mandatory. This procedure was time consuming and impacted significantly on study activation timelines. In addition, during the entire period of study activation, there was a change in the therapeutic landscape and management of patients with endocrine sensitive MBC. Introduction and approval of CDK 4/6 inhibitors and PI3K-inhibitors in combination with endocrine treatment was recommended by ESMO and national guidelines as the first choice of treatment for first line therapy for HR-positive MBC. This new information was approached with a further Amendment to the clinical protocol. Spain left the project in June 2018 due to funding shortage after enrollment of three out of 10 preplanned patients; France and Italy completed accrual as planned; Germany enrolled eight patients but stopped accrual in 2019. The total number of enrolled patients was 147 out of 310 planned patients (47.4%) of which 88 in Italy, 48 in France, eight in Germany, and three in Spain. Overall accrual was stopped on 22nd January 2021 due to [^18^F]- FES shortage as the primary reason but also for substantially exceeding the project deadlines with consequent exhaustion of the funds allocated for the project.

## Discussion

There has been an increasing interest in molecular imaging by experimental radiotracers in oncology. Especially with the approval and introduction into clinical practice of effective but extremely expensive new targeted agents, the sustainability of the cost of these medications is rapidly becoming an emergency in health policies in the EU. For this reason, personalized medicine is quickly becoming an unmet need also in health economics. The possibility of treatment individualization, based on the detection by molecular imaging of the *in vivo* activity of drug targets and pathways, in addition to molecular assessment on tissue biopsies, may represent the missing step in delivering the right (expensive) drug to the patient with the highest benefit. This will also optimize treatment in those patients who are not likely to respond, thus sparing ineffective therapies. However, this process requires the formal validation of these new molecular tracers in well-designed translational trials. These types of trials are particularly difficult in terms of “sustainability” as well. Additionally, the costs of new radiotracers and of high-quality research are high, so dedicated funding is needed and can only be achieved through academic grants. Pharmaceutical companies are of course reluctant in principle to fund research project aimed at treatment individualization if the approval for a wider indication has already been achieved. Academic trials therefore become fundamental for promoting trials which are not attractive to big pharma. To this context, our project could provide additional evidence on the performance of these innovative techniques in treatment individualization based on the results of a randomized clinical trial. Directive 2001/20/EC intended to harmonize clinical trial application but in practice, the regulatory requirements are not really harmonized due to specific regulatory requirements and variability across EU Members States (MS) in particular for innovative drugs including Radio-pharmaceuticals. This problem will be overcome by European Regulation 536/2014 that will come into force on 31/01/2022 as the new submission procedure will be centralized. An existing pathway that could has been used to facilitate the process is the Voluntary Harmonization Procedure (VHP), an Initiative of the Clinical Trials Facilitation Group to gain experience in the practical work within the ideas of a “CT- regulation” and to offer an option for sponsors and Member States to achieve harmonized multi-national clinical trials and share workload. From a regulatory perspective, taking into account that the ET-FES trial involves an experimental drug ([^18^F]-FES), without Market authorization in the EU, the approval process was timely completed at EC level in all the participating institutions but time to CA approval was unexpectedly different in the various countries; this was probably due to a different interpretation of the rules, guidelines and requirements from each local CA, indicating the absence of really harmonized procedures as requested by the 2001/20/EC Directive. Furthermore, additional causes of delay were encountered: in Italy, the critical issue concerned the administrative procedures to activate this type of international EU projects, requiring a suboptimal time span, in order to satisfy all the legal aspects on contracts by public bodies, in Germany some radioprotection concerns further delayed authorization. These issues and timelines need to be considered and solved, when applying for EU calls where the allowed project duration is 3 years. Performing a clinical trial requires a dedicated infrastructure to deal with the administrative and regulatory requirements; for this reason, it may not be accessible for Academia or Collaborative Groups, to deal with national and even more with an international clinical trial. Administrative aspects at local institutions, ECs, ministry of health and other involved agencies were very difficult to approach. This was also due to the fact that in addition to the ERANET programme rules, each center has to obey national laws and regulations concerning Clinical Research. Indeed, in our experience, each regulatory competent authority asked for different modifications of the study protocol, mainly IMPD and Investigator Brochure of [^18^F]-FES, which we had to solve before starting of the project. It was very difficult and time consuming to align all the changes requested by the national competent authorities; for this reason time elapsed and the study became “old” as new drugs came on the market. Nevertheless, with a strong commitment from all partners we finally overcame almost all the bureaucratic, administrative and regulatory problems and the study was finally activated. Unfortunately the project had only a 3 year duration and the costs could no longer be allocated to the European project. The implementation of a national network for co-operation in clinical science would facilitate multidisciplinary clinical research, as well as provide guidelines and models of good practices for national support infrastructures. Hub and spoke networks of oncological centers, along with a multidisciplinary approach, is the winning strategy to offer additional skills and expertise through the involvement of different specialists not always heavily involved in clinical research. In this project, in particular, nuclear medicine is a crucial aspect and the standardization of image acquisition protocols is one of the most important requirements among network participating centers. It's important that the hub of the network provides a dedicated infrastructure to harmonize the roles and responsibilities, facilitate the communication between the trial promoter and each center/ethical committee/national and local competent authority, supervise the timing of each step and provide help in those centers requiring expertise and support for specific duties related to the trial. Furthermore, it should produce and diffuse specific guidelines to enhance the comparability of data acquired by molecular imaging and to boost molecular imaging so that it becomes a standard diagnostic modality in future clinical medicine and research (7, 8).

Main barriers to speeding up the process and the possible solutions can be categorized in three main areas:

1. **Administrative complexity**:
  a. **Approval at European level**—an experienced grant office is needed to speed up the submission in particular for the national funding aspects.
  b. **EC and CA approval**—even if the scope of European Directive was to align all study documents, in practical there is eterogeneity; documents should be centralized and made available for all researchers with a forum for academic investigators to share their issues. The regulatory competencies should be shared and implemented in collaboration with the relevant international agencies and ethics committees. Scientific advice and support for non-commercial sponsors should be provided with practical support for clinical trial submissions through an easy to-follow flow chart and guidelines.
  c. **Local feasibility approval**—for administrative and economic items there is an urgent need for a centralized management; for radioprotection, the new Directive 2013/59/Euratom should facilitate but an alignment by all Competent Authorities on this topic is needed.
2. **Heterogeneous staff training**: Developing training, education and knowledge in clinical research for whole the multidisciplinary team will further a “culture” of clinical research and create a professional network of experienced people.
3. **Infrastructure support**: The presence of adequate personnel within regulatory and legal office, grant office, radio pharmacy, clinical trial office (study coordinator and biostatisticians) is the winning strategy to reach the goal. All these expertise are involved from clinical protocol writing to final data analysis. National institutions should promote the development of clinical research infrastructures with the above competences and support functions organized in networks of research units and investigators.

A summary of main barriers details and possible solutions are reported in Table 3. All these barriers and realistic timelines must be taken into account when evaluating project feasibility, before applying for an European grant.

**Table 3:** Main barrier details and possible solutions.

**Issue**	**Proposed solution**	**Comments**
Documents preparation on a national level cause misalignment	The implementation of a national network for co-operation will facilitate multidisciplinary clinical research, as well as provide guidelines and models of good practices for national support infrastructures.	Even if the scope of Directive 2001/20/CE is to align all study documents, in practical there is eterogeneity
Lack of experienced personnel	Develop training, education and knowledge on clinical research to all the multidisciplinary team will develop a “culture” of clinical research and a professional network of experienced people	All these expertise are involved from clinical protocol writing to final data analysis.
Each National Principal Investigator submitted the study to its own EC/IRB and CA.	Scientific advice and support for non-commercial sponsors should be provided with practical support for trial submissions; all the informations should be available with a forum for academic investigators to share their issues.	New Regulation 536/2014 will hopefully overcome this issue by centralizing trial submission
Approval timelines for EC and AC approvals are legally defined according to European Directive; the different submission timing caused different approval timing.	Same timing of submission to EC and CA	Directive foresees a national approval that will be superseded by Regulation 536/2014
Different CA objections on quality IMPD; CA approval was unexpectedly different in the various countries. Need for Substantial Amendments to align country specific documentation	CA opinions should be the same; regulatory competencies should be shared and implemented in collaboration with the relevant international agencies and ethics committees.	This is caused by different quality guidelines interpretations
Confidential quality data not shared by Company with the Sponsor; this process is not under Sponsor control and responsibility.	Clearly define confidential data policy in terms of ownership and responsibilities between Sponsor and the Company.	The incoming Regulation 536/2014 may probably overcome this issue with co-sponsorship.
Contracts and local administrative item is very time consuming. Every Country was funded by the responsible national funding organization according to their specific regulations	Single contract, centralized economic management	
Time consuming procedures related to the high heterogeneity between National radioprotection competent authorities	Competent Authorities on radioprotection should be aligned at an European level	

## Data Availability Statement

The original contributions presented in the study are included in the article/supplementary material, further inquiries can be directed to the corresponding author/s.

## Ethics Statement

The studies involving human participants were reviewed and approved by IRST and Romagna Ethics Committee (CEIIAV). The patients/participants provided their written informed consent to participate in this study.

## Author Contributions

MM and TD contributed for regulatory aspects, acquisition of data, and drafting the manuscript. NH, EB, RW, JC, AP-G, and CS contributed to the study design and conception of the study. JA, AP, FM, HI, LB, and ON contributed to study design. AG contributed to the study design, conception of the study, and project coordination. VD contributed to IMPD data. MP, AA, MI, DC, and GR contributed to project starting procedures. All authors provided substantial input in the study design, critical revision of the manuscript, and read and approved the final manuscript.

## Funding

This research received grants from ERANET JTC 2011.

## Conflict of Interest

The authors declare that the research was conducted in the absence of any commercial or financial relationships that could be construed as a potential conflict of interest.

## Publisher's Note

All claims expressed in this article are solely those of the authors and do not necessarily represent those of their affiliated organizations, or those of the publisher, the editors and the reviewers. Any product that may be evaluated in this article, or claim that may be made by its manufacturer, is not guaranteed or endorsed by the publisher.
